# Ever-Young Sex Chromosomes in European Tree Frogs

**DOI:** 10.1371/journal.pbio.1001062

**Published:** 2011-05-17

**Authors:** Matthias Stöck, Agnès Horn, Christine Grossen, Dorothea Lindtke, Roberto Sermier, Caroline Betto-Colliard, Christophe Dufresnes, Emmanuel Bonjour, Zoé Dumas, Emilien Luquet, Tiziano Maddalena, Helena Clavero Sousa, Iñigo Martinez-Solano, Nicolas Perrin

**Affiliations:** 1 Department of Ecology and Evolution, University of Lausanne, Lausanne, Switzerland; 2 Department of Biology, University of Fribourg, Fribourg, Switzerland; 3 Umweltmikrobiologie, EAWAG, Dübendorf, Switzerland; 4 UMR 5023 Ecology of Fluvial Hydrosystems, Bât. Darwin C, Université Lyon, Villeurbanne, France; 5 Gordevio, Switzerland; 6 Perpetuo Socorro, Puerto Santa María (Cádiz), Spain; 7 Instituto de Investigación en Recursos Cinegéticos (UCLM-CSIC-JCCM), Ciudad Real, Spain; University of California Santa Barbara, United States of America

## Abstract

Non-recombining sex chromosomes are expected to undergo evolutionary decay, ending up genetically degenerated, as has happened in birds and mammals. Why are then sex chromosomes so often homomorphic in cold-blooded vertebrates? One possible explanation is a high rate of turnover events, replacing master sex-determining genes by new ones on other chromosomes. An alternative is that X-Y similarity is maintained by occasional recombination events, occurring in sex-reversed XY females. Based on mitochondrial and nuclear gene sequences, we estimated the divergence times between European tree frogs (*Hyla arborea*, *H. intermedia*, and *H. molleri*) to the upper Miocene, about 5.4–7.1 million years ago. Sibship analyses of microsatellite polymorphisms revealed that all three species have the same pair of sex chromosomes, with complete absence of X-Y recombination in males. Despite this, sequences of sex-linked loci show no divergence between the X and Y chromosomes. In the phylogeny, the X and Y alleles cluster according to species, not in groups of gametologs. We conclude that sex-chromosome homomorphy in these tree frogs does not result from a recent turnover but is maintained over evolutionary timescales by occasional X-Y recombination. Seemingly young sex chromosomes may thus carry old-established sex-determining genes, a result at odds with the view that sex chromosomes necessarily decay until they are replaced. This raises intriguing perspectives regarding the evolutionary dynamics of sexually antagonistic genes and the mechanisms that control X-Y recombination.

## Author Summary

Non-recombining sex chromosomes, such as the Y chromosome, are expected to degenerate over evolutionary times because they accumulate deleterious mutations that cannot be corrected by recombination with a pristine copy. In most cold-blooded vertebrates, such as frogs, however, sex chromosomes are undifferentiated. Why is that so? On the one hand, the “high-turnover” hypothesis holds that these sex chromosomes are regularly replaced before they had time to decay. On the other hand, the “fountain-of-youth” hypothesis posits that they are regularly rejuvenated by X-Y recombination in sex-reversed XY females. Here, we show that three species of tree frogs that diverged more than 5.4 million years ago share the same pair of undifferentiated sex chromosomes. Although male recombination stopped before species divergence, X and Y alleles show no differentiation, and cluster by species, not gametologs. We conclude that their sex chromosome homomorphy is not due to a recent turnover but is maintained over long evolutionary times by occasional recombination. Such rare episodes of X-Y recombination are expected to have long-lasting consequences on the evolution of sex chromosomes and sex antagonistic genes.

## Introduction

The highly decayed Y chromosome of mammals results from an evolutionary process that started some 170 million years ago (mya), when a new masculinizing gene (*SRY*) first appeared on an autosome [1]–[2]. Recombination then stopped in males in the vicinity of this new sex-determining gene, presumably to preserve epistatic interactions with sexually antagonistic mutations [3]. Genes that happened to be trapped in the non-recombining segment accumulated deleterious mutations under the combined forces of genetic drift, selective sweeps, background selection, and Muller's ratchet [4]. Similar processes are thought to have occurred in birds [5], where females are the heterogametic sex, carrying a degenerated, non-recombining (W) chromosome. The seemingly ineluctable decay induced by the lack of recombination has led to the suggestion that sex chromosomes are “born to be destroyed” [6], though a prevailing opinion is that gene loss slows down over time [4] and that gene content might still show rapid evolution in old sex chromosomes [7].

However, in sharp contrast with birds and mammals, decay and differentiation are rarely observed in cold-blooded vertebrates. Sex chromosomes have been described as homomorphic in about 96% of amphibians studied so far [8], and similar numbers are found in fishes [9]. Even recognizing that seemingly homomorphic chromosomes might show some differentiation at finer scales, the contrast with warm-blooded vertebrates is striking. Why is that so? Two alternative models propose contrasting explanations. On the one hand, the “high-turnover” hypothesis suggests that master sex-determining genes are regularly replaced by new ones, so that the non-recombining segments that later evolve around the new sex-determining gene do not have enough time to degenerate [10]. Direct evidence for recent turnover events is indeed accumulating [11]–[13], with different heterogametic systems found in closely related species, or even in populations from the same species [14]. However, it is not clear whether such events occur often enough to account for the overwhelming prevalence of sex-chromosome homomorphy. Phylogenetic analyses of amphibians have identified only seven heterogametic transitions during the evolutionary history of this species-rich group [15], which certainly leaves enough time for the Y or W to diverge, even assuming that some turnovers did not affect heterogamety.

On the other hand, the “fountain-of-youth” hypothesis [16] holds that sex-chromosome integrity can be maintained over long evolutionary times by occasional recombination in XY females. Sex-reversal experiments have shown that sex differences in the recombination patterns of several vertebrate and invertebrate species depend on phenotypic sex, not on genotype [17]–[20]. The sex-reversed XY females of medaka fish display female-specific recombination patterns, while sex-reversed XX males show the characteristic male absence of recombination [21]–[22]. Similar patterns occur in frogs [23]. As sex reversal occasionally occurs in ectotherms (due to the temperature dependence of physiological processes underlying sex determination [24]–[26]), the ensuing recombination in XY females should oppose Muller's ratchet and prevent the evolutionary decay of sex chromosomes.

### Model System and Specific Predictions

Here we use European tree frogs to test contrasting predictions from these two models. All Eurasian tree frogs have homomorphic sex chromosomes [27]. Male heterogamety was first evidenced in *Hyla arborea* by sex differences in the allelic distribution of microsatellite markers [28]–[29]. Mapping linkage groups through sibship analyses identified nine sex-linked markers, which all revealed complete absence of male recombination, despite overlapping X-Y allelic distributions [30]. Similarity between gametologs was further confirmed by cDNA sequences of a sex-linked transcription cofactor: apart from some frame-preserving indels in polyglutamine repeat tracts (which are known for their high rate of slippage mutation), the X and Y copies showed no single base substitutions over 2,400 bp, including >800 synonymous sites [31].

**Figure 1:**
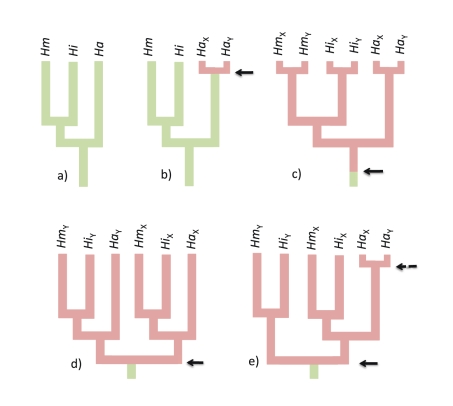
Expected gene genealogies under different evolutionary scenarios. The focal gene is localized either on an autosome (green) or on a sex chromosome (red) in *H. arborea* (*Ha*), *H. intermedia* (*Hi*), or *H. molleri* (*Hm*). Arrows indicate turnovers in sex-determination systems. (a) Reference genealogy for an autosomal or mitochondrial marker. (b) In *H. arborea*, the marker lies on a proto sex chromosome recently derived from an autosome. Sex linkage is restricted to *H. arborea*, and genealogy conforms to species genealogy. (c) The marker is on ancestral sex chromosomes and thus sex-linked in all three species, but its genealogy still conforms to species genealogy due to occasional X-Y recombination. (d) The marker is on ancestral sex chromosomes and thus sex-linked in all three species, but due to absence of X-Y recombination, alleles cluster according to gametologs, not species. Within gametologs, gene genealogy conforms to species genealogy. (e) In *H. arborea*, the marker lies on a proto sex chromosome recently derived from an ancestral sex chromosome (dashed arrow), such that *Ha_Y_* clusters with the ancestral *Ha_X_*. The marker is sex linked in all three species, but in the sister group of *H. arborea*, alleles cluster according to gametolog, not species. Note that a similar genealogy would result from local gene conversion (see Figure 1 in [35]).

Is this striking X-Y similarity maintained by occasional recombination, or does it result from a recent turnover, followed by the rapid loss of male recombination? To test between these two alternatives, we combined investigations on gene genealogies and recombination patterns in two species from the sister clade to *H. arborea*, namely the Italian *H. intermedia* and the Iberian *H. molleri* [32]. The recent turnover model predicts that the sex chromosomes will differ between *H. arborea* and its sister-group species (as is observed, e.g., in medakas, sticklebacks, or tilapias [11]–[13]). Markers shown to be sex-linked in *H. arborea* are thus expected to display both autosomal localization and normal male recombination in the sister species, while their genealogies (Figure 1b) should conform to the species genealogy (Figure 1a). If, however, the sex chromosomes are ancestral, these markers should display sex linkage and absence of male recombination in all three species (Figure 1c–e). Furthermore, under the X-Y-recombination model, gene genealogies should conform to species genealogy (so that alleles cluster according to species; Figure 1c), while the opposite outcome (clustering by gametologs) would occur if X-Y recombination definitely stopped before species divergence (Figure 1d). Note that if a recent turnover occurred on ancestral sex chromosomes (with, e.g., the *H. arborea* proto-Y derived from the ancestral X) [33], then markers in sister-group species should also be sex-linked but alleles should cluster by gametologs (Figure 1e).

## Results

### Species Divergence Times

**Figure 2:**
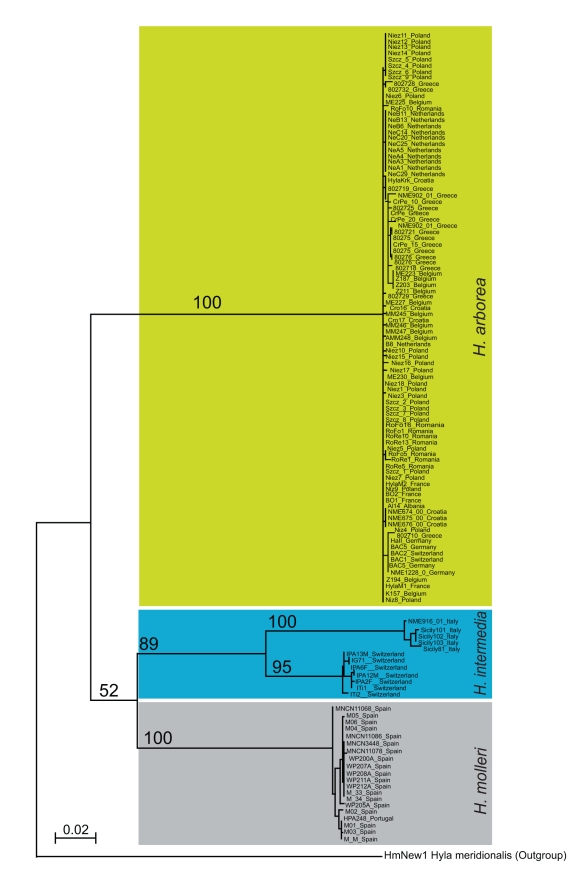
Maximum-likelihood phylogeny for tree frog *cytochrome b* lineages. The divergence time between mtDNA *cytochrome b* lineages of *H. arborea* and sister-group species (complete sequences of ca 1000 bp, multiple samples across species geographic ranges) averages 7.1 my (2.3–15.8 my 95% HPDI). Origin of samples and GenBank accession numbers are provided in Table S5.

Phylogenetic analyses of mitochondrial and nuclear genes showed that *Hyla arborea* diverged from the sister species (*H. intermedia* and *H. molleri*) during the late Miocene, namely around the Messinian salinity crisis. Estimates point to lower Messinian (7.1 my, 95% HPDI 2.3 – 15.8 my) for the mtDNA *cytochrome b* (Figure 2) and upper Messinian (5.4 my, 95% HPDI 1.4 – 12.3 my) for intronic sequences of the nuclear *Fibrinogen alpha*.

### Sex-Specific Linkage Maps

**Figure 3:**
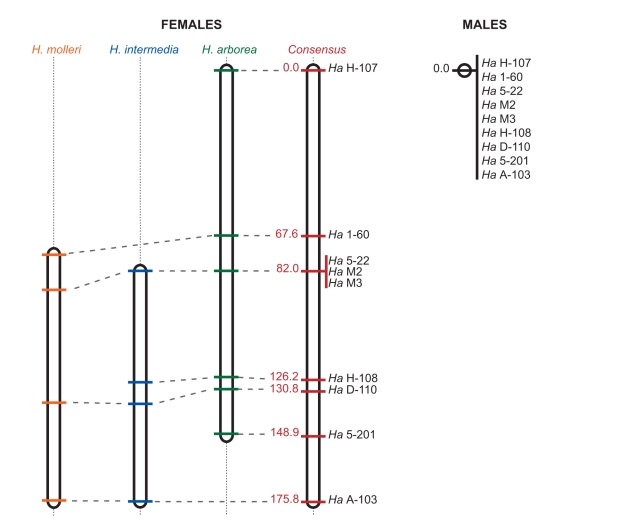
Recombination maps for sex-linked markers . The complete absence of recombination in males (right) contrasts sharply with the high recombination rates found in females (left). Lengths are given in cM units for the consensus map, and correspondences are provided graphically for species-specific maps. In each case the map is the one with highest likelihood, except that for *H. intermedia*, ranking third but with a log-likelihood very close to (and not significantly lower than) the first one (−120.79 versus −119.71).

Several of the nine microsatellites found to be sex-linked in *H. arborea* could be cross-amplified (six in *H. intermedia* and six in *H. molleri*). We genotyped a total of 111 families from the three species, each comprising a mating pair and an average of 20 offspring, plus a few additional non-mating adults (Table S1). Sibship analyses revealed shared synteny and complete linkage in males (Table S2a–d). In females, by contrast, pairwise recombination rates were very high (most of them between 0.30 and 0.50). These patterns did not differ between species (Morton M-test [34]), so that the three datasets could be pooled to produce a consensus map (Figure 3). Parsimony implies that male recombination stopped before the species diverged. This provides sufficient time to allow detectable sequence differentiation between non-recombining X and Y chromosomes, as otherwise found at nuclear and mitochondrial sequences (Figure 2).

### Sex Linkage

The linkage groups in Figure 3 map to sex chromosomes in all three species. Despite the scarcity of sex-diagnostic alleles, sex linkage could be established on two grounds. First, significant sex differences in allelic frequencies were found at several loci in all species (Text S1). Second, sibship analyses and multilocus associations provided evidence for the coexistence of several different non-recombining Y haplotypes in natural populations (Text S2 and Table S3). In all cases, autosomal localization (Figure 1b) could be rejected with high confidence.

### Patterns of X-Y Similarities

**Figure 4:**
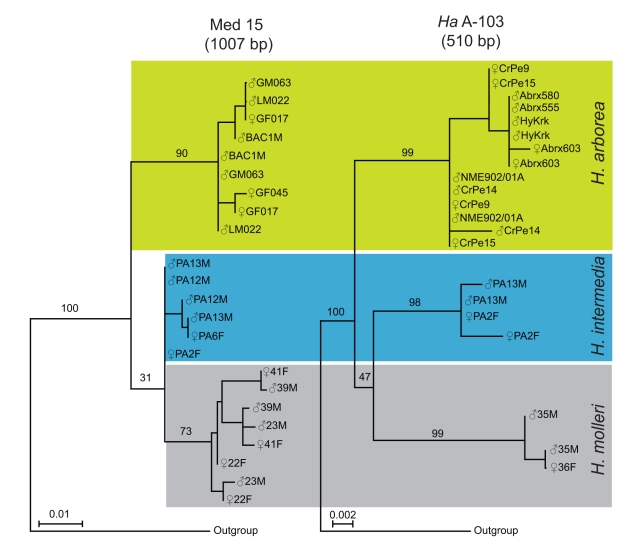
Gene genealogies for two sex-linked loci. The transcription cofactor *HaMed15* (left, ca. 1 kb sequences with two introns and two exons, including the marker *Ha* 5–22) and the non-coding *Ha* A-103 (right, ca. 510 bp sequences) are 93.8 cM apart on the female recombination map (Figure 3). For both markers, the X and Y alleles (marked with the same label when amplified from the same male) cluster by species, not by gametolog. Bootstrap values are higher for the non-coding *Ha* A-103 (≥98%) than for the highly conserved transcription cofactor *HaMed15* (≤90%), and higher for *H. arborea* than for species from its sister group.

Finally, we found higher X-Y similarity within the three species than between them, however we assessed it. First, size differences between conspecific X and Y alleles were smaller than between alleles randomly sampled at the same locus from different species (Figure S1), implying shorter coalescence times. Second, patterns of cross-amplifications depended on species more than on gametologs (Table S4), implying higher primer-sequence similarity between conspecific sex chromosomes than heterospecific gametologs. Third, the X and Y sequences of two sex-linked loci, chosen for their distant localization on the sex chromosomes (93.8 cM in the female consensus map) clustered according to species, not gametologs (Figure 4).

## Discussion

We conclude that all three species studied inherited the same pair of XY sex chromosomes from a common ancestor but that, despite absence of recombination in males, Y chromosomes show higher sequence similarities and overlap in allele frequency distributions with conspecific X chromosomes than with allospecific Y chromosomes. Hence, sex-chromosome homomorphy in *H. arborea* does not result from a recent turnover event, from either an autosome (Figure 1b) or an ancestral sex chromosome (Figure 1e). Local gene conversion between X and Y chromosomes (Figure 1e) occasionally occurs in mammals [35] but cannot parsimoniously account for the large-scale X-Y similarity found in all markers and species, with respect not only to the sequence data (Figure 4) but also to the patterns of allelic sizes (Figure S1) and cross-amplifications (Table S4) at genotyping markers. Our data thus support occasional X-Y recombination (Figure 1c), occurring either in males or in sex-reversed XY females.

The maintenance of a potential for X-Y recombination over evolutionary times contrasts sharply with our failure to measure any recombination in males (Figure 3), raising important issues regarding the underlying mechanisms [36]. Recombination in *H. arborea* males is suppressed on all sex-linked markers and drastically repressed on autosomes [29], arguing against local mechanisms such as inversions [37]. Genome-wide effects with phenotypic-sex dependence are likely to stem from meiotic or epigenetic processes [38]. Meiosis in frogs occurs at very different times and under different physiological conditions in male and female germ cell lineages [39], while imprinted genomic regions in humans are known to display large sex differences in recombination rates [40]–[41].

Our findings have important implications for the evolutionary dynamics of sex chromosomes. Given the high rate of female recombination documented here (Figure 3), a single event of sex reversal is expected to generate a wide diversity of new Y haplotypes. In the absence of male recombination, the fittest ones (i.e., those purged of the deleterious mutations that accumulate during periods of non-recombination, but still having the male-beneficial alleles at sexually antagonistic loci) should be sorted out by natural or sexual selection and spread among natural populations within a few generations. This interplay of recombination and selective sweeps might account for the significant differences in allelic frequencies, despite low sequence differentiation, between X and Y chromosomes. Phylogeographic studies of Y haplotypes over the range of *H. arborea*, which recently expanded into Western Europe from a West-Balkanic glacial refugium [32], might help in uncovering historical signatures of such events. Signatures might also be found at the genomic level, with peaks of X-Y divergence in the vicinity of sex-determining or sex-antagonistic loci, which might be detected by looking at the coalescence times of neutral markers [42].

From our results, seemingly “young” sex chromosomes may harbor old sex-determining genes. The sex-determination system shared by these tree frog species may thus considerably predate their divergence. It will be interesting to study species further apart in the phylogeny (e.g., *H. savignyi*, *H. meridionalis*, or *H. japonica* [32]). In a wider perspective, similar investigations focusing on sister groups of species from other taxa, sharing the same pair of undifferentiated sex chromosomes, might allow estimates of the extent to which X-Y recombination contributes to the overwhelming prevalence of sex-chromosome homomorphy among cold-blooded vertebrates.

The fountain-of-youth and high-turnover hypotheses, however, are not to be seen as exclusive alternatives. The same mechanisms responsible for sex reversal and X-Y recombination (e.g., temperature shift stemming from a range expansion) may also generate turnover events via sex-ratio selection [26], and the homomorphy maintained by occasional recombination may create favorable conditions for sex chromosome turnovers from other mechanisms, such as sex-antagonistic selection [43].

## Materials and Methods

### Animal Sampling and DNA Extraction

The resource pedigree consisted of 2,863 individuals from 111 known family groups, each including a mother, father, and an average of 20 offspring per family (Table S1). Mating pairs caught in amplexus in the field were allowed to spawn; then buccal cells were sampled [44] before release. A few additional crosses between *H. arborea* populations were produced in the lab (Table S1). Clutches (one per mating pair) were maintained in the laboratory until tadpoles had grown enough to allow tissue sampling (tip of tail). Buccal swabs and tissues were stored at −20°C before analysis. DNA was extracted using a QIAGEN DNeasy Tissue Kit following the manufacturer's protocol with few additional steps [30] or using the BioSprint robotic workstation (QIAGEN). DNA was eluted in a 200 µl volume (QIAGEN Buffer AE) and stored at −18°C.

### Microsatellite Primers, Amplifications, and Scoring

We used published primer sequences [28]–[30],[45]–[46] except for *Ha* M2 and *Ha* M3, which, together with *Ha* 5–22, correspond to poly-Glutamine chains within different exons of the sex-linked gene *HaMed15*, and for which we designed primers based on the published X and Y sequences (GenBank EU276188 and EU276189) [31]. *Ha* M2 (F: 5′ GCC TGT TGA GCT GCT TGC 3′; R: 5′ GGG CAG TGC AAG CTC AGC 3′) ranges from 100 to 120 bp and has a complex motif including CAG, CAA, and GCA repeats. *Ha* M3 (F: 5′ CTG GTT TTG CTG TTG CTG AA 3′; R: 5′ TCA AGT CAC CCA GCA GAA TG 3′) has a size ranging from 175 to 185 bp and a complex motif including CAG and CAA repeats. Multiplex PCRs were carried out for the two loci in a total reaction volume of 10 µl containing 0.2 µM of each primer, 0.6× of Multiplex PCR Master Mix (QIAGEN), and 3 µl of extracted DNA. PCR amplifications were performed on the GeneAmp PCR Systems 2700 and 9700 (Perkin Elmer, Norwalk, CT) according to the following thermal conditions: initial denaturation at 95°C for 15 min followed by 32 cycles of denaturation at 94°C for 30 s, annealing at 58°C for 1 min 30 s, elongation at 72° for 1 min, and then a final elongation step at 60°C for 30 min. The same conditions were used to successfully amplify these two markers in *H. molleri* and *H. intermedia*.

For other primers, PCR reactions were conducted in two independent multiplex reactions (QIAGEN) co-amplifying up to six microsatellites [30],[45], except for marker *Ha* 1–60 in *H. molleri* and markers *Ha* 5–22, *Ha* H-108, and *Ha* D-110 in *H. intermedia*, which were amplified individually as follows: 10 µl reaction volume each containing 0.25 mM dNTP, 0.5 µM of each primer, 1× QIAGEN PCR Buffer (with MgCl2 15 mM), 0.2 mM MgCl2 (0.5 mM MgCl2 for *Ha* D-110 and no MgCl2 for *Ha* 5-22 and *Ha* H-108), 1× QIAGEN Q-Solution, between 0.03 U and 0.1 U QIAGEN Taq, and between 1 and 3 µl of extracted DNA. PCR reactions were performed on GeneAmp PCR Systems 2700 and 9700 (Perkin Elmer, Norwalk, CT) according to the following thermal profiles: initial denaturation at 95°C for 15 min (94° for 5 min for individual amplification with QIAGEN Taq) followed by 32–35 cycles at 94°C for 45 s (QIAGEN Taq: 40–45 cycles), annealing at 58°C for 45 s (60°C for *Ha* H-108 in *H. intermedia* and *Ha* 1-60 in *H. molleri*), elongation at 72°C for 1 min, and a final elongation step at 60°C for 30 min (QIAGEN Taq: 75°C for 5 min). PCR products were analyzed on an automated sequencer (ABI Prism 3100 Genetic Analyzer, Applied Biosystems). Allele sizes and genotypes were determined using GeneMapper 4.0 (Applied Biosystems) followed by manual proofreading. In order to confirm homology, alleles from each microsatellite locus were cloned and sequenced in all three species.

### Population-Genetics and Linkage Analyses

Allele frequencies in males and females were calculated with FSTAT 2.9.3.2 [47]. Linkage analyses were performed with CRIMAP 5.0 [48] using the same procedures as in [30]. Heterogeneity in recombination rates among populations and species was tested for each available marker interval with Morton's M-test [34]. In absence of heterogeneity, sample sets were pooled with the option *merge*.

### Amplification, Cloning, and Alignment of Sequences

The mitochondrial *cytochrome b* gene was amplified with primers L0 and H1046 [32]. PCR products were sequenced in both directions, visualized on an ABI 3730 sequencer, and aligned with SEQUENCHER 4.9.

To amplify ca. 545 bp of intron 1 of *Fibrinogen A*, *alpha-*polypeptide, we used two primers (MVZ47: 5′_AGTGAAAGATACAGTCACAGTGCTAGG_3′; MVZ48: 5′_GGAGGATATCAGCACAGTCTAAAAAG_3′) and a protocol developed by Jason B. Mackenzie in the Museum of Vertebrate Zoology (University of California, Berkeley). PCR were carried out in 12.5 µl reactions containing 7.55 µl H_2_O, 1.25 µl of PCR buffer including 1.5 mM MgCl_2_, 0.1 µl of dNTPs, 0.1 µl Taq QIAGEN, 0.75 µl of each primer having a concentration of 10 µM, and 2 µl of genomic DNA with a concentration of 20 ng/µl. For subsequent cloning, two of such reactions from each individual were pooled to increase volume. The PCR protocol followed a “touch-up” approach with 10 cycles of increasing annealing temperatures (55°C to 60°C) by 0.5 degrees each cycle (with 30 s at 95°C, 30 s at annealing temperature, and 45 s at 72°C), followed by 25 cycles with 30 s at 94°C, 30 s at 56°C, and 45 s at 72°C, and a final extension of 7 min at 72°C.

The sex-linked gene *HaMed15* (ca. 1 kb fragments including 2 exons and 2 introns) was amplified with primers Ha 5-22F (5′-TTACAGCAACAGCAAATGG-3′) and p984R (5′_CGAGTATGCTTAATAGCTAATGCTA_3′). PCRs (94°C 1.5 min, 37×(94°C 45 s, 55°C 45 s, 72°C 1 min), 72°C 5 min) were carried out in 25 µl reaction volumes containing 17.75 µl H_2_O, 2.5 µl of PCR buffer including 1.5 mM MgCl_2_, 1.1 µl of a solution containing 2.0 mM MgCl_2_, 0.25 µl of dNTPs, 0.4 µl Taq QIAGEN, 0.5 µl of each primer having a concentration of 10 µM, and 2 µl of genomic DNA with a concentration of 10 ng/µl.

The sex-linked non-coding marker *Ha A-103* was amplified (ca. 510 bp) with primers Ha A-103F1 (5′_GCCTAGAAATGTGCAGTGATC_3′) and Ha A-103R2 (5′_TGGAAAGTTTGCCCATTCAT_3′). PCRs (94°C 1.5 min, 40×(94°C 45 s, 50°C 54 s, 72°C 40 s), 72°C 5 min) were carried out in 25 µl reaction volumes containing 19 µl H_2_O, 2.5 µl of PCR buffer including 1.5 mM MgCl_2_, 0.25 µl of dNTPs, 0.25 µl Taq QIAGEN, 0.5 µl of each primer having a concentration of 10 µM, and 2 µ µl of genomic DNA with a concentration of 10 ng/µl.

For all nuclear markers, PCR products were cloned using the pGEM-easy vector system (Promega). Concentrations were first quantified (NanoDrop ND-1000 spectrometer) and adjusted to 25 ng/µl. We mixed 1.5 µl of template, 0.075 µl of vector (50 ng/µl), 2.5 µl 2× ligation buffer, 0.5 µl T4 ligase, and 0.425 µl water and ligated overnight (10°C). Transformations were carried out by incubating a mixture of 2.5 µl ligation mix and 12–25 µl JM109 High Efficiency competent cells for 20 min on ice and then heat-shocking them for 45 s at 42°C. Transformed cells were recovered in SOC medium for 1 h 30 min; 80–100 µl of cell suspension was applied to LB agar plates supplied with Ampicillin/IPTG/X-Gal. After incubation (18 h, 37°C), templates from a number of 10–12 white colonies were amplified with forward and reverse vector-specific primers M13. Nested vector-specific primers T7 and SP6 (Promega) were used as sequencing primers. All clones were sequenced in both directions and visualized on an ABI 3730 sequencer and aligned with SEQUENCHER 4.9. For all sex-linked markers we sequenced 10–12 clones from each individual to minimize the risk of allelic dropout; alleles were aligned and screened for singletons to correct for PCR error. Sequences included in phylogenetic analyses are thus represented by multiple clones each. GenBank accession numbers for *HaMed15* sequences are JF317989 to JF318012; for the microsatellite-containing sequence *Ha* A103: JF318144 to JF318169; for intron 1 of the *Fibrinogen A*, *alpha*-polypeptide: JF318013 to JF318047. Those for *cytochrome b* are provided in Table S5.

### Phylogenetic Analyses

Maximum likelihood (ML) phylogenies were generated with *PhyML* 3.0 [49] using the GTR model for *cytochrome b* and HKY model for sex-linked (*Ha* A-103, *HaMed15*) and autosomal (*Fibrinogen alpha*) nuclear markers. For each case, we chose a BioNJ tree as a starting tree and used the combined subtree pruning and regrafting (SPR) plus nearest neighbor interchange (NNI) options for tree improvement. All other parameters were set as default (http://atgc.lirmm.fr/phyml/). Bootstrap values were based on 1,000 resampled datasets.

### Molecular Dating

Divergence times were estimated assuming an uncorrelated exponential relaxed molecular clock. For the mitochondrial *cytochrome b* gene, we assumed a normal distribution of priors for the substitution rate, with mean 0.01 my^−1^ (±0.007 SD) [50]–[51], and a GTR plus gamma model of sequence evolution (Modeltestserver 1.0). We used a Yule tree prior (constant speciation rate per lineage) as most appropriate for species-level divergences [52]. DNA sequence data were analyzed both with and without codon partition, with different partitions for codons 1+2 and 3 (results turned out to be very robust regarding partitioning). For the *Fibrinogen alpha* gene (intron 1) we followed the same approach but used a HKY plus Gamma model of sequence evolution (Modeltestserver 1.0) and normal prior distributions for substitution rates with mean values ranging 0.001 to 0.002 my^−1^ [53]. Analyses were run for 20 Mio generations each and repeated to ensure stability of estimates.

## Supporting Information

Figure S1 Distribution of allelic size differences at sex-linked loci. Conspecific X and Y alleles differ on average by 6.26 bp (red vertical line), obtained as the size difference between the two alleles expressed in a male (average over loci and species; *n* = 649 in total). The distribution (vertical bars) of mean size differences between 649 pairs of alleles randomly sampled at the same loci from different species (100,000 replicates) averages 9.24 bp (±0.35 SD). From this distribution, an average difference of 6.26 bp or less has a probability *p*<0.00001 to occur by chance.
(EPS)

Table S1 Sampling localities for families. Given are the species, locality with coordinates, numbers of adult males (Nm), adult females (Nf), families (Na), and offspring (No). In *H. arborea*, two series of families (respectively 9 and 8) resulted from lab crosses between individuals from different populations (Cheylas-Laissaud and Lavigny-Flaach).
(DOC)

Table S2 Matrices of recombination rates. Male values (blue) are above diagonal, and female values (green) below diagonal. Cells are empty when one of the two markers involved could not be amplified for this sex/species, and labeled as NA if the rate could not be assessed, as occurs when markers are not simultaneously polymorphic within parents. Markers *Ha* M2 and *Ha* M3 are not included, being strictly linked to *Ha* 5–22 (all are parts of the gene *HaMed15*). The consensus matrix was obtained by merging family files from all three species. Corresponding LOD scores are available upon request.
(DOC)

Table S3 Genotypes of adult tree frogs. (a) 13 males (top) and 13 females (bottom) from the *Lavigny* population of *H. arborea*. The four Y haplotypes (marked in color) identified through sibship analyses and sex-specific allelic frequencies differ only at *Ha* 1–60. Null alleles are coded by a star (*). (b) 24 males (top) and 24 females (bottom) from the *Piazzogna* population of *H. intermedia*. The five Y haplotypes (marked in color) identified through sibship analyses and sex-specific allelic frequencies differ at three loci (*Ha* 5–22, *Ha* D-110, and *Ha* A-103). Null alleles are coded by a star (*). (c) 13 males (top) and 2 females (bottom) from the *Cantera* population of *H. molleri*. A minimum of two Y haplotypes (marked in color) can be recognized based on sibship analyses and sex-specific allelic frequencies. Presumed null alleles are coded by a star (*). In three males no product could be amplified at *Ha* D-110 (marked as NA).
(DOC)

Table S4 Matrix of cross-amplification patterns per locus, species, and gametolog. In 14 instances no product could be amplified in any individual (entry = 0), but in only two cases did the amplification patterns differ between conspecific X and Y (namely for *Ha* D-110 and *Ha* A-103 in *H. arborea*). Random permutations of the matrix (100,000 replicates) show that such a low number (two cases or less) has a probability *p*<0.0002 to occur by chance.
(DOC)

Table S5 Sample information for sequences used in mtDNA cytb phylogenies. Provided are identification numbers with species name and voucher information, locality coordinates, and GenBank accession numbers.
(DOC)

Text S1 Sex differences in allelic frequencies.
(DOC)

Text S2 Non-recombining Y haplotypes in males.
(DOC)

## Abbreviations

cM: centiMorgan
HPDI: high probability density interval
ML: maximum likelihood
SRY: sex-determining region of Y chromosome
