# Supplementary material for: Ever-Young Sex Chromosomes in European Tree Frogs
Source: PLoS Biol. 2011 May 17;9(5):e1001062. doi: 10.1371/journal.pbio.1001062 (PMC3100596; doi:10.1371/journal.pbio.1001062)
Supplement: Table S1 — Sampling localities for families. Given are the species, locality with coordinates, numbers of adult males (Nm), adult females (Nf), families (Na), and offspring (No). In H. arborea, two series of families (respectively 9 and 8) resulted from lab crosses between individuals from different populations (Cheylas-Laissaud and Lavigny-Flaach). (DOC) [file pbio.1001062.s002.doc]

**Table S1**

| **Species** | **Population** | **Geographic coordinates** | | **Nm** | **Nf** | **Na** | **No** | **TOTAL** |
| --- | --- | --- | --- | --- | --- | --- | --- | --- |
| *H. molleri* | Cantera | 40°12'34.02"N | 4°38'43.01"W | 13 | 2 | 1 | 51 | 66 |
|  | La Dehesa | 40°13'52.10"N | 4°36'17.06"W | 2 | 0 | 0 | 0 | 2 |
|  | El Tiemblo | 40°24'40.50"N | 4°28'05.80"W | 6 | 0 | 0 | 0 | 6 |
|  | Valdemanco | 40°51'12.42"N | 3°38'41.68"W | 5 | 5 | 4 | 200 | 210 |
|  | Fuente Sotillo | 40°16'06.55"N | 4°34'25.02"W | 3 | 6 | 3 | 130 | 139 |
|  | **Total** |  |  | **29** | **13** | **8** | **381** | **423** |
| *H. intermedia* | Piazzogna (TI) | 46°08'10.16''N | 8°49'14.20"E | 24 | 24 | 24 | 693 | 741 |
|  | **Total** |  |  | **24** | **24** | **24** | **693** | **741** |
| *H. arborea* | Cheylas | 45°22'53''N | 5°59'48''E | 22 | 31 | 22 | 388 | 441 |
|  | Laissaud | 45°28'18''N | 6°3'51''E | 10 | 1 | 1 | 16 | 27 |
|  | Cheylas-Laissaud |  |  |  |  | 9 | 191 | 191 |
|  | Planches | 45°44'20''N | 5°21'7''E | 12 | 12 | 12 | 298 | 322 |
|  | Lavigny | 46°30'9.00''N | 6°25'10.38''E | 15 | 19 | 13 | 215 | 249 |
|  | Camp Romain | 46°31'18.48''N | 6°21'13.46''E | 14 | 14 | 14 | 209 | 237 |
|  | Flaach | 47°34'55.24''N | 8°36'07.23''E | 6 | 2 |  |  | 8 |
|  | Lavigny-Flaach |  |  |  |  | 8 | 224 | 224 |
|  | **Total** |  |  | **79** | **79** | **79** | **1541** | **1699** |
