# Supplementary material for: Ever-Young Sex Chromosomes in European Tree Frogs
Source: PLoS Biol. 2011 May 17;9(5):e1001062. doi: 10.1371/journal.pbio.1001062 (PMC3100596; doi:10.1371/journal.pbio.1001062)
Supplement: Table S2 — Matrices of recombination rates. Male values (blue) are above diagonal, and female values (green) below diagonal. Cells are empty when one of the two markers involved could not be amplified for this sex/species, and labeled as NA if the rate could not be assessed, as occurs when markers are not simultaneously polymorphic within parents. Markers Ha M2 and Ha M3 are not included, being strictly linked to Ha 5–22 (all are parts of the gene HaMed15). The consensus matrix was obtained by merging family files from all three species. Corresponding LOD scores are available upon request. (DOC) [file pbio.1001062.s003.doc]

**Table S2**

| ***a) H. arborea*** | |  |  |  |  |  |  |
| --- | --- | --- | --- | --- | --- | --- | --- |
|  | ***Ha* H-107** | ***Ha* 1-60** | ***Ha* 5-22** | ***Ha* H-108** | ***Ha* D-110** | ***Ha* 5-201** | ***Ha* A-103** |
| ***Ha* H-107** |  | 0.00 | 0.00 | 0.00 | 0.00 | 0.00 | 0.00 |
| ***Ha* 1-60** | 0.44 |  | 0.00 | 0.00 | 0.00 | 0.00 | 0.00 |
| ***Ha* 5-22** | 0.41 | 0.14 |  | 0.00 | 0.00 | 0.00 | 0.00 |
| ***Ha* H-108** | NA | 0.48 | 0.3 |  | 0.00 | 0.00 | 0.00 |
| ***Ha* D-110** | 0.44 | 0.4 | 0.42 | 0.04 |  | 0.00 | 0.00 |
| ***Ha* 5-201** | 0.5 | 0.43 | 0.49 | 0.26 | 0.17 |  | 0.00 |

| ***b) H. intermedia*** | |  |  |  |  |  |  |
| --- | --- | --- | --- | --- | --- | --- | --- |
|  | ***Ha* H-107** | ***Ha* 1-60** | ***Ha* 5-22** | ***Ha* H-108** | ***Ha* D-110** | ***Ha* 5-201** | ***Ha* A-103** |
| ***Ha* H-107** |  |  |  |  |  |  |  |
| ***Ha* 1-60** |  |  |  |  |  |  |  |
| ***Ha* 5-22** |  |  |  | 0.00 | 0.00 |  | 0.00 |
| ***Ha* H-108** |  |  | 0.32 |  | 0.00 |  | 0.00 |
| ***Ha* D-110** |  |  | 0.5 | NA |  |  | 0.00 |
| ***Ha* 5-201** |  |  |  |  |  |  |  |
| ***Ha* A-103** |  |  | 0.43 | 0.33 | 0.50 |  |  |

| ***c) H. molleri*** | |  |  |  |  |  |  |
| --- | --- | --- | --- | --- | --- | --- | --- |
|  | ***Ha* H-107** | ***Ha* 1-60** | ***Ha* 5-22** | ***Ha* H-108** | ***Ha* D-110** | ***Ha* 5-201** | ***Ha* A-103** |
| ***Ha* H-107** |  |  |  |  |  |  |  |
| ***Ha* 1-60** |  |  | 0.00 |  | 0.00 |  | 0.00 |
| ***Ha* 5-22** |  | 0.14 |  |  | 0.00 |  | 0.00 |
| ***Ha* H-108** |  |  |  |  |  |  |  |
| ***Ha* D-110** |  | 0.45 | 0.36 |  |  |  | 0.00 |
| ***Ha* 5-201** |  |  |  |  |  |  |  |
| ***Ha* A-103** |  | 0.41 | 0.41 |  | 0.34 |  |  |

| ***d) Hyla* consensus** | | |  |  |  |  |  |
| --- | --- | --- | --- | --- | --- | --- | --- |
|  | ***Ha* H-107** | ***Ha* 1-60** | ***Ha* 5-22** | ***Ha* H-108** | ***Ha* D-110** | ***Ha* 5-201** | ***Ha* A-103** |
| ***Ha* H-107** |  | 0.00 | 0.00 | 0.00 | 0.00 | 0.00 | 0.00 |
| ***Ha* 1-60** | 0.44 |  | 0.00 | 0.00 | 0.00 | 0.00 | 0.00 |
| ***Ha* 5-22** | 0.41 | 0.14 |  | 0.00 | 0.00 | 0.00 | 0.00 |
| ***Ha* H-108** | NA | 0.48 | 0.32 |  | 0.00 | 0.00 | 0.00 |
| ***Ha* D-110** | 0.44 | 0.41 | 0.39 | 0.04 |  | 0.00 | 0.00 |
| ***Ha* 5-201** | 0.50 | 0.43 | 0.49 | 0.26 | 0.17 |  | 0.00 |
| ***Ha* A-103** |  | 0.41 | 0.41 | 0.33 | 0.35 |  |  |
