# Supplementary material for: Ever-Young Sex Chromosomes in European Tree Frogs
Source: PLoS Biol. 2011 May 17;9(5):e1001062. doi: 10.1371/journal.pbio.1001062 (PMC3100596; doi:10.1371/journal.pbio.1001062)
Supplement: Table S3 — Genotypes of adult tree frogs. (a) 13 males (top) and 13 females (bottom) from the Lavigny population of H. arborea. The four Y haplotypes (marked in color) identified through sibship analyses and sex-specific allelic frequencies differ only at Ha 1–60. Null alleles are coded by a star (*). (b) 24 males (top) and 24 females (bottom) from the Piazzogna population of H. intermedia. The five Y haplotypes (marked in color) identified through sibship analyses and sex-specific allelic frequencies differ at three loci (Ha 5–22, Ha D-110, and Ha A-103). Null alleles are coded by a star (*). (c) 13 males (top) and 2 females (bottom) from the Cantera population of H. molleri. A minimum of two Y haplotypes (marked in color) can be recognized based on sibship analyses and sex-specific allelic frequencies. Presumed null alleles are coded by a star (*). In three males no product could be amplified at Ha D-110 (marked as NA). (DOC) [file pbio.1001062.s004.doc]

**Table S3a**

| ID | *Ha* H-107 | | *Ha* 1-60 | | *Ha* 5-22 | | *Ha* M2 | | *Ha* M3 | | *Ha* H-108 | | *Ha* D-110 | | *Ha* 5-201 | | *Ha* A-103 | |
| --- | --- | --- | --- | --- | --- | --- | --- | --- | --- | --- | --- | --- | --- | --- | --- | --- | --- | --- |
| *H.arb* M1023 A10 | 267 | 267 | 120 | 130 | 230 | 236 | 112 | 121 | 184 | 175 | 250 | 252 | 342 | *** | 236 | 233 | *** | 237 |
| *H.arb* M1381 A4 | 267 | 267 | 120 | 130 | 230 | 236 | 112 | 121 | 184 | 175 | 250 | 252 | 342 | *** | 236 | 233 | *** | 237 |
| *H.arb* M1193 A2 | 267 | 267 | 122 | 130 | 230 | 236 | 112 | 121 | 184 | 175 | 250 | 252 | 334 | *** | 242 | 233 | *** | 237 |
| *H.arb* M1419 A6 | 269 | 267 | 122 | 137 | 230 | 236 | 112 | 121 | 184 | 175 | 250 | 252 | 338 | *** | 242 | 233 | *** | 237 |
| *H.arb* M1136 A17 | 267 | 267 | 116 | 137 | 230 | 236 | 112 | 121 | 184 | 175 | 250 | 252 | 338 | *** | 242 | 233 | *** | 237 |
| *H.arb* M1002 A1 | 267 | 267 | 120 | 139 | 230 | 236 | 112 | 121 | 184 | 175 | 250 | 252 | 338 | *** | 242 | 233 | *** | 237 |
| *H.arb* M1369 A3 | 267 | 267 | 120 | 139 | 230 | 236 | 100 | 121 | 184 | 175 | 250 | 252 | 342 | *** | 242 | 233 | *** | 237 |
| *H.arb* M1457 A9 | 269 | 267 | 116 | 139 | 230 | 236 | 112 | 121 | 184 | 175 | 250 | 252 | 334 | *** | 242 | 233 | *** | 237 |
| *H.arb* M1403 A5 | 267 | 267 | 120 | 139 | 230 | 236 | 112 | 121 | 184 | 175 | 250 | 252 | 338 | * | 242 | 233 | *** | 237 |
| *H.arb* M1431 A7 | 269 | 267 | 116 | 139 | 230 | 236 | 112 | 121 | 184 | 175 | 250 | 252 | 342 | *** | 242 | 233 | *** | 237 |
| *H.arb* M1115 A16 | 267 | 267 | 122 | 139 | 230 | 236 | 112 | 121 | 184 | 175 | 250 | 252 | 338 | * | 242 | 233 | *** | 237 |
| *H.arb* M1158 A18 | 269 | 267 | 120 | 139 | 230 | 236 | 112 | 121 | 184 | 175 | 250 | 252 | 338 | * | 242 | 233 | *** | 237 |
| *H.arb* M1435 A8 | 267 | 267 | 120 | 141 | 230 | 236 | 112 | 121 | 184 | 175 | 250 | 252 | 345 | *** | 236 | 233 | *** | 237 |
|  |  |  |  |  |  |  |  |  |  |  |  |  |  |  |  |  |  |  |
| *H.arb* F1024 A10 | 267 | 267 | 116 | 122 | 230 | 230 | 112 | 112 | 184 | 184 | 250 | 250 | 338 | 360 | 233 | 236 | *** | *** |
| *H.arb* F1382 A4 | 267 | 269 | 120 | 122 | 230 | 230 | 112 | 112 | 184 | 184 | 250 | 250 | 338 | 338 | 236 | 242 | *** | *** |
| *H.arb* F1192 A2 | 267 | 269 | 120 | 122 | 230 | 230 | 112 | 112 | 184 | 184 | 250 | 250 | 342 | 353 | 230 | 242 | *** | *** |
| *H.arb* F1420 A6 | 269 | 269 | 120 | 120 | 230 | 230 | 100 | 112 | 184 | 184 | 250 | 250 | 338 | 353 | 242 | 242 | *** | *** |
| *H.arb* F1137 A17 | 267 | 267 | 120 | 122 | 230 | 230 | 112 | 112 | 184 | 184 | 250 | 250 | 334 | 338 | 242 | 242 | *** | *** |
| *H.arb* F1001 A1 | 267 | 269 | 120 | 120 | 230 | 230 | 100 | 112 | 184 | 184 | 250 | 250 | 338 | 353 | 230 | 242 | *** | *** |
| *H.arb* F1370 A3 | 267 | 267 | 120 | 122 | 230 | 230 | 112 | 112 | 184 | 184 | 250 | 250 | 338 | 338 | 236 | 242 | *** | *** |
| *H.arb* F1458 A9 | 267 | 269 | 116 | 122 | 230 | 230 | 112 | 112 | 184 | 184 | 250 | 250 | 338 | 338 | 230 | 242 | *** | *** |
| *H.arb* F1404 A5 | 267 | 267 | 116 | 120 | 230 | 230 | 100 | 112 | 184 | 184 | 250 | 250 | 338 | 338 | 242 | 242 | *** | *** |
| *H.arb* F1432 A7 | 267 | 267 | 122 | 122 | 230 | 230 | 112 | 112 | 184 | 184 | 250 | 250 | 334 | 342 | 236 | 242 | *** | *** |
| *H.arb* F1114 A16 | 267 | 269 | 120 | 122 | 230 | 230 | 112 | 112 | 184 | 184 | 250 | 250 | 338 | 338 | 233 | 242 | *** | *** |
| *H.arb* F1159 A18 | 267 | 267 | 122 | 122 | 230 | 230 | 112 | 112 | 184 | 184 | 250 | 250 | 338 | 353 | 230 | 242 | *** | *** |
| *H.arb* F1436 A8 | 267 | 267 | 122 | 124 | 230 | 230 | 112 | 112 | 184 | 184 | 250 | 250 | 338 | 338 | 236 | 242 | *** | *** |

**Table S3b**

| ID | *Ha* 5-22 | | *Ha* M2 | | *Ha* M3 | | *Ha* H-108 | | *Ha* D-110 | | *Ha* A-103 | |
| --- | --- | --- | --- | --- | --- | --- | --- | --- | --- | --- | --- | --- |
| *H.int* M27 A4 | 221 | 218 | 120 | 120 | 182 | 182 | 220 | 250 | * | 485 | 225 | 225 |
| *H.int* M47 A14 | 218 | 218 | 120 | 120 | 173 | 182 | 226 | 250 | * | 485 | 223 | 225 |
| *H.int* M57 A19 | 218 | 218 | 120 | 120 | 182 | 182 | 250 | 250 | * | 485 | 223 | 225 |
| *H.int* M69 A25 | 221 | 218 | 120 | 120 | 182 | 182 | 214 | 250 | * | 485 | 223 | 225 |
| *H.int* M49 A15 | 221 | 221 | 120 | 120 | 182 | 182 | 250 | 250 | * | 490 | 223 | 225 |
| *H.int* M1 A1 | 221 | 221 | 120 | 120 | 182 | 182 | 250 | 250 | 485 | 485 | 225 | 223 |
| *H.int* M23 A2 | 218 | 221 | 120 | 120 | 173 | 182 | 250 | 250 | * | 485 | 227 | 223 |
| *H.int* M31 A6 | 218 | 221 | 120 | 120 | 182 | 182 | 250 | 250 | * | 485 | 223 | 223 |
| *H.int* M33 A7 | 221 | 221 | 120 | 120 | 182 | 182 | 220 | 250 | * | 485 | 223 | 223 |
| *H.int* M35 A8 | 221 | 221 | 120 | 120 | 182 | 182 | 250 | 250 | * | 485 | 223 | 223 |
| *H.int* M37 A9 | 218 | 221 | 120 | 120 | 182 | 182 | 250 | 250 | * | 485 | 223 | 223 |
| *H.int* M43 A12 | 218 | 221 | 120 | 120 | 182 | 182 | 250 | 250 | * | 485 | 223 | 223 |
| *H.int* M51 A16 | 221 | 221 | 120 | 120 | 182 | 182 | 214 | 250 | * | 485 | 225 | 223 |
| *H.int* M61 A21 | 218 | 221 | 120 | 120 | 182 | 182 | 214 | 250 | * | 485 | 223 | 223 |
| *H.int* M65 A23 | 221 | 221 | 120 | 120 | 182 | 182 | 250 | 250 | 515 | 485 | 223 | 223 |
| *H.int* M67 A24 | 221 | 221 | 120 | 120 | 182 | 182 | 226 | 250 | 490 | 485 | 223 | 223 |
| *H.int* M53 A17 | 221 | 221 | 120 | 120 | 182 | 182 | 250 | 250 | * | 519 | 223 | 227 |
| *H.int* M59 A20 | 221 | 221 | 120 | 120 | 182 | 182 | 214 | 250 | * | 519 | 227 | 227 |
| *H.int* M63 A22 | 218 | 221 | 120 | 120 | 182 | 182 | 250 | 250 | * | 519 | 223 | 227 |
| *H.int* M25 A3 | 221 | 221 | 120 | 120 | 182 | 182 | 220 | 250 | * | 528 | 223 | 227 |
| *H.int* M29 A5 | 221 | 221 | 120 | 120 | 182 | 182 | 250 | 250 | * | 528 | 223 | 227 |
| *H.int* M39 A10 | 221 | 221 | 120 | 120 | 182 | 182 | 214 | 250 | * | 528 | 223 | 227 |
| *H.int* M41 A11 | 218 | 221 | 120 | 120 | 182 | 182 | 214 | 250 | 485 | 528 | 223 | 227 |
| *H.int* M45 A13 | 221 | 221 | 120 | 120 | 182 | 182 | 250 | 250 | * | 528 | 223 | 227 |
|  |  |  |  |  |  |  |  |  |  |  |  |  |
| *H.int* F2 A1 | 218 | 221 | 120 | 120 | 182 | 182 | 214 | 250 | * | 485 | 223 | 223 |
| *H.int* F24 A2 | 218 | 221 | 120 | 120 | 182 | 182 | 250 | 250 | * | * | 223 | 225 |
| *H.int* F26 A3 | 218 | 221 | 120 | 120 | 182 | 182 | 216 | 226 | * | * | 223 | 223 |
| *H.int* F28 A4 | 218 | 218 | 120 | 120 | 182 | 182 | 220 | 250 | * | * | 225 | 227 |
| *H.int* F30 A5 | 218 | 221 | 120 | 120 | 182 | 182 | 214 | 220 | * | * | 223 | 223 |
| *H.int* F32 A6 | 221 | 221 | 120 | 120 | 182 | 182 | 214 | 226 | * | * | 223 | 225 |
| *H.int* F34 A7 | 218 | 221 | 120 | 120 | 182 | 182 | 226 | 250 | * | * | 223 | 223 |
| *H.int* F35 A8 | 218 | 218 | 120 | 120 | 182 | 182 | 250 | 250 | * | 515 | 223 | 223 |
| *H.int* F38 A9 | 218 | 218 | 120 | 120 | 182 | 182 | 226 | 250 | * | * | 223 | 223 |
| *H.int* F40 A10 | 218 | 221 | 120 | 120 | 182 | 182 | 226 | 226 | * | * | 223 | 223 |
| *H.int* F42 A11 | 218 | 221 | 120 | 120 | 182 | 182 | 250 | 250 | * | 485 | 223 | 227 |
| *H.int* F44 A12 | 218 | 218 | 120 | 120 | 182 | 182 | 214 | 250 | * | * | 223 | 223 |
| *H.int* F46 A13 | 218 | 221 | 120 | 120 | 182 | 182 | 214 | 250 | * | 515 | 223 | 225 |
| *H.int* F48 A14 | 218 | 221 | 120 | 120 | 182 | 182 | 250 | 250 | * | * | 223 | 223 |
| *H.int* F50 A15 | 221 | 221 | 120 | 120 | 182 | 182 | 214 | 214 | * | * | 225 | 225 |
| *H.int* F48 A16 | 218 | 224 | 120 | 120 | 182 | 182 | 250 | 250 | * | * | 223 | 223 |
| *H.int* F A17 | 218 | 221 | 120 | 120 | 182 | 182 | 226 | 250 | * | * | 223 | 223 |
| *H.int* F48 A19 | 218 | 221 | 120 | 120 | 182 | 182 | 222 | 222 | * | 515 | 223 | 223 |
| *H.int* F50 A20 | 218 | 221 | 120 | 120 | 182 | 182 | 214 | 250 | * | * | 223 | 223 |
| *H.int* F62 A21 | 218 | 218 | 120 | 120 | 182 | 182 | 220 | 250 | * | * | 223 | 227 |
| *H.int* F50 A22 | 218 | 221 | 120 | 120 | 182 | 182 | 214 | 250 | * | * | 223 | 225 |
| *H.int* F66 A23 | 218 | 221 | 120 | 120 | 182 | 182 | 214 | 226 | * | * | 223 | 225 |
| *H.int* F68 A24 | 218 | 218 | 120 | 120 | 182 | 182 | 226 | 250 | * | * | 223 | 223 |
| *H.int* F70 A25 | 221 | 221 | 120 | 120 | 182 | 182 | 226 | 250 | * | * | 223 | 223 |

**Table S3c**

| ID | *Ha* 1-60 | | *Ha* 5-22 | | *Ha* M2 | | *Ha* M3 | | *Ha* D-110 | | *Ha* A-103 | |
| --- | --- | --- | --- | --- | --- | --- | --- | --- | --- | --- | --- | --- |
| *H.moll* 13M | 140 | 140 | 236 | 233 | 120 | 120 | 182 | 170 | 584 | 626 | 232 | 235 |
| *H.moll* 05M | 149 | 140 | 224 | 233 | 120 | 120 | 182 | 170 | 626 | 626 | 232 | 235 |
| *H.moll* 06M | 116 | 140 | 230 | 233 | 120 | 120 | 182 | 170 | 580 | 626 | 232 | 235 |
| *H.moll* 07M | 149 | 140 | 224 | 233 | 120 | 120 | 182 | 170 | 584 | 626 | 232 | 235 |
| *H.moll* 08M | 149 | 140 | 227 | 233 | 120 | 120 | 182 | 170 | 363 | 626 | 237 | 235 |
| *H.moll* 10M | 131 | 140 | 227 | 233 | 120 | 120 | 182 | 170 | 363 | 626 | 232 | 235 |
| *H.moll* 11M | 140 | 140 | 224 | 233 | 120 | 120 | 182 | 170 | 694 | 626 | 241 | 235 |
| *H.moll* 04M | 149 | 142 | 230 | 224 | 120 | 120 | 182 | 182 | 580 | * | 232 | 229 |
| *H.moll* 09M | 116 | 142 | 230 | 224 | 120 | 120 | 182 | 182 | NA | NA | 232 | 229 |
| *H.moll* 12M | 149 | 142 | 230 | 224 | 120 | 120 | 182 | 182 | 580 | * | 232 | 229 |
| *H.moll* 14M | 149 | 142 | 230 | 224 | 120 | 120 | 182 | 182 | NA | NA | 227 | 229 |
| *H.moll* 17M | 149 | 142 | 230 | 224 | 120 | 120 | 182 | 182 | NA | NA | 227 | 229 |
| *H.moll* 23M A1 | 149 | 142 | 230 | 224 | 120 | 120 | 182 | 182 | 626 | * | 227 | 229 |
|  |  |  |  |  |  |  |  |  |  |  |  |  |
| *H.moll* 22F A1 | 149 | 142 | 227 | 224 | 120 | 120 | 182 | 182 | 626 | 626 | 232 | 219 |
| *H.moll* 18F | 149 | 131 | 227 | 224 | 120 | 120 | 182 | 182 | 584 | 626 | 237 | 232 |
