# Supplementary material for: Ever-Young Sex Chromosomes in European Tree Frogs
Source: PLoS Biol. 2011 May 17;9(5):e1001062. doi: 10.1371/journal.pbio.1001062 (PMC3100596; doi:10.1371/journal.pbio.1001062)
Supplement: Table S4 — Matrix of cross-amplification patterns per locus, species, and gametolog. In 14 instances no product could be amplified in any individual (entry = 0), but in only two cases did the amplification patterns differ between conspecific X and Y (namely for Ha D-110 and Ha A-103 in H. arborea). Random permutations of the matrix (100,000 replicates) show that such a low number (two cases or less) has a probability p<0.0002 to occur by chance. (DOC) [file pbio.1001062.s005.doc]

**Table S4**

|  | *H.moll* X | *H.moll* Y | *H.int* X | *H.int* Y | *H.arb* X | *H.arb* Y |
| --- | --- | --- | --- | --- | --- | --- |
| *Ha* H-107 | 0 | 0 | 0 | 0 | 1 | 1 |
| *Ha* 1-60 | 1 | 1 | 0 | 0 | 1 | 1 |
| *Ha* 5-22 | 1 | 1 | 1 | 1 | 1 | 1 |
| *Ha* M2 | 1 | 1 | 1 | 1 | 1 | 1 |
| *Ha* M3 | 1 | 1 | 1 | 1 | 1 | 1 |
| *Ha* H-108 | 0 | 0 | 1 | 1 | 1 | 1 |
| *Ha* D-110 | 1 | 1 | 1 | 1 | 1 | 0 |
| *Ha* 5-201 | 0 | 0 | 0 | 0 | 1 | 1 |
| *Ha* A-103 | 1 | 1 | 1 | 1 | 0 | 1 |
