# Supplementary material for: Ever-Young Sex Chromosomes in European Tree Frogs
Source: PLoS Biol. 2011 May 17;9(5):e1001062. doi: 10.1371/journal.pbio.1001062 (PMC3100596; doi:10.1371/journal.pbio.1001062)
Supplement: Table S5 — Sample information for sequences used in mtDNA cytb phylogenies. Provided are identification numbers with species name and voucher information, locality coordinates, and GenBank accession numbers. (DOC) [file pbio.1001062.s006.doc]

| **Table S5. Sample information for sequences used in mtDNA cytb phylogenies** | | | | | |
| --- | --- | --- | --- | --- | --- |
| **Sample ID**  **(Fig. 2)** | **Species** | **Specimen Voucher** | **Locality** | **Lat**  **Lon** | **GenBank Acc. -Number Cyt *b*** |
| 802710  Greece | *Hyla arborea* | NHMC  80.2.7.10 | Greece, Lesvos isl. | 39.260 N  26.281 E | JF318048 |
| 802718  Greece | *Hyla arborea* | NHMC  80.2.7.18 | Greece, Peloponnesos, Strofilia | 38.165 N  21.381 E | JF318049 |
| 802719  Greece | *Hyla arborea* | NHMC  80.2.7.19 | Greece, Peloponnesos, Strofilia | 38.165 N  21.381 E | JF318050 |
| 802721  Greece | *Hyla arborea* | NHMC 80.2.7.21 | Greece, Crete, Kroussonas, (N central Crete on Mount Ida) | 35.2335 N  24.9831 E | FJ226867 |
| 802725  Greece | *Hyla arborea* | NHMC  80.2.7.25 | Greece, Peloponnesos, Strofilia | 38.165 N  21.381 E | JF318051 |
| 802728  Greece | *Hyla arborea* | NHMC  80.2.7.28 | Greece, Thessaly - Pertouli, conifer forest | 39.566 N  21.508 E | JF318052 |
| 802729  Greece | *Hyla arborea* | NHMC  80.2.7.29 | Greece, Thessaly - Pertouli, conifer forest | 39.566 N  21.508 E | JF318053 |
| 802732  Greece | *Hyla arborea* | NHMC  80.2.7.32 | Greece, Thessaly - Pertouli, conifer forest | 39.566 N  21.508 E | JF318054 |
| 80275  Greece | *Hyla arborea* | NHMC 80.2.7.5 | Greece, Crete, Lassithi plateau (East Crete) | 35.192 N  25.4314 E | FJ226868 |
| 80276  Greece | *Hyla arborea* | NHMC 80.2.7.6 | Greece, Crete, Lassithi plateau (East Crete) | 35.192 N  25.4314 E | FJ226920 |
| Al14  Albania | *Hyla arborea* | - | Albania, Tirana | 41.322 N  19.87 E | JF318055 |
| B8  Netherlands | *Hyla arborea* | - | Netherlands, Beekvliet | 52.0658 N  6.2839 E | JF318056 |
| BAC1  Switzerland | *Hyla arborea* | - | Switzerland, near village Lavigny | 46.5024 N  6.4195 | FJ226861 |
| BAC2  Switzerland | *Hyla arborea* | - | Switzerland, near village Lavigny | 46.5024 N  6.4195 E | FJ226862 |
| BAC5  Germany | *Hyla arborea* | - | Germany, NRW, near Münster | 51.95 N  7.7 E | FJ226863 |
| BAC6  Germany | *Hyla arborea* | - | Germany, NRW, near Münster | 51.95 N  7.7 E | FJ226864 |
| BO1  France | *Hyla arborea* | - | France, La Dombes | 46.01 N  4.94 | FJ226865 |
| BO2  France | *Hyla arborea* | - | France, La Dombes | 46.01 N  4.94 E | FJ226866 |
| Cro16  Croatia | *Hyla arborea* | - | Croatia, Bacinska Lakes | 43.0711 N  17.4160 E | JF318057 |
| Cro17  Croatia | *Hyla arborea* | - | Croatia, Bacinska Lakes | 43.0711 N  17.4160 E | JF318058 |
| CrPe_10  Greece | *Hyla arborea* | - | Greece, Crete, Kiperas Pond | 35.1756 N  25.3044 | JF318059 |
| CrPe_15  Greece | *Hyla arborea* | - | Greece, Crete, Kiperas Pond | 35.1756 N  25.3044 E | JF318060 |
| CrPe_20  Greece | *Hyla arborea* | - | Greece, Crete, Kiperas Pond | 35.1756 N  25.3044 | JF318061 |
| CrPe  Greece | *Hyla arborea* | - | Greece, Crete, Kiperas Pond | 35.1756 N  25.3044 E | JF318062 |
| HaII  Germany | *Hyla arborea* | - | Germany, Saxony, Papitz near Leipzig | 51.38 N  12.24 | FJ226921 |
| HylaKrk  Croatia | *Hyla arborea* | NME A 1758/10 | Croatia, Krk island | 45.1747 N  14.5593 E | JF318063 |
| HylaM1  France | *Hyla arborea* | - | France, Bretagne, Monterfil | 48.0468 N  -1.9714 | FJ226824 |
| HylaM2  France | *Hyla arborea* | - | France, Bretagne, Monterfil | 48.0468 N  -1.9714 | FJ226825 |
| K157  Belgium | *Hyla arborea* | - | Belgium, Knokke | 50.9772 N  2.8024 | JF318064 |
| ME223  Belgium | *Hyla arborea* | - | Belgium, Maaseik | 51.0948 N  5.7939 E | JF318065 |
| ME225  Belgium | *Hyla arborea* | - | Belgium, Maaseik | 51.0948 N  5.7939 E | JF318066 |
| ME227  Belgium | *Hyla arborea* | - | Belgium, Maaseik | 51.0948 N  5.7939 E | JF318067 |
| ME230  Belgium | *Hyla arborea* | - | Belgium, Maaseik | 51.0948 N  5.7939 E | JF318068 |
| MM245  Belgium | *Hyla arborea* | - | Belgium, Maasmechelen, Maaswinhel | 50.9616 N  5.7006 E | JF318069 |
| MM246  Belgium | *Hyla arborea* | - | Belgium, Maasmechelen, Maaswinhel | 50.9616 N  5.7006 E | JF318070 |
| MM247  Belgium | *Hyla arborea* | - | Belgium, Maasmechelen, Maaswinhel | 50.9616 N  5.7006 E | JF318071 |
| MM248  Belgium | *Hyla arborea* | - | Belgium, Maasmechelen, Maaswinhel | 50.9616 N  5.7006 E | JF318072 |
| NeA1  Netherlands | *Hyla arborea* | - | Netherlands | 52.2308 N  6.5549 E | JF318073 |
| NeA3  Netherlands | *Hyla arborea* | - | Netherlands | 52.2308 N  6.5549 E | JF318074 |
| NeA4  Netherlands | *Hyla arborea* | - | Netherlands | 52.2308 N  6.5549 E | JF318075 |
| NeA5  Netherlands | *Hyla arborea* | - | Netherlands | 52.2308 N  6.5549 E | JF318076 |
| NeB11  Netherlands | *Hyla arborea* | - | Netherlands | 52.0658 N  6.2839 E | JF318077 |
| NeB13  Netherlands | *Hyla arborea* | - | Netherlands | 52.0658 N  6.2839 E | JF318078 |
| NeB6  Netherlands | *Hyla arborea* | - | Netherlands | 52.0658 N  6.2839 E | JF318079 |
| NeC14  Netherlands | *Hyla arborea* | - | Netherlands | 52.064 N  6.2841 E | JF318080 |
| NeC20  Netherlands | *Hyla arborea* | - | Netherlands | 52.064 N  6.2841 E | JF318081 |
| NeC25  Netherlands | *Hyla arborea* | - | Netherlands | 52.064 N  6.2841 | JF318082 |
| NeC29  Netherlands | *Hyla arborea* | - | Netherlands | 52.064 N  6.2841 E | JF318083 |
| Niez1  Poland | *Hyla arborea* | - | Poland, Niezgoda | 51.5153 N  17.0508 E | JF318084 |
| Niez10  Poland | *Hyla arborea* | - | Poland, Niezgoda | 51.5153 N  17.0508 | JF318085 |
| Niez11  Poland | *Hyla arborea* | - | Poland, Niezgoda | 51.5153 N  17.0508 | JF318086 |
| Niez12  Poland | *Hyla arborea* | - | Poland, Niezgoda | 51.5153 N  17.0508 E | JF318087 |
| Niez13  Poland | *Hyla arborea* | - | Poland, Niezgoda | 51.5153 N  17.0508 | JF318088 |
| Niez14  Poland | *Hyla arborea* | - | Poland, Niezgoda | 51.5153 N  17.0508 E | JF318089 |
| Niez15  Poland | *Hyla arborea* | - | Poland, Niezgoda | 51.5153 N  17.0508 E | JF318090 |
| Niez16  Poland | *Hyla arborea* | - | Poland, Niezgoda | 51.5153 N  17.0508 E | JF318091 |
| Niez17  Poland | *Hyla arborea* | - | Poland, Niezgoda | 51.5153 N  17.0508 E | JF318092 |
| Niez18  Poland | *Hyla arborea* | - | Poland, Niezgoda | 51.5153 N  17.0508 E | JF318093 |
| Niez3  Poland | *Hyla arborea* | - | Poland, Niezgoda | 51.5153 N  17.0508 E | JF318094 |
| Niez4  Poland | *Hyla arborea* | - | Poland, Niezgoda | 51.5153 N  17.0508 E | JF318095 |
| Niez5  Poland | *Hyla arborea* | - | Poland, Niezgoda | 51.5153 N  17.0508 E | JF318096 |
| Niez6  Poland | *Hyla arborea* | - | Poland, Niezgoda | 51.5153 N  17.0508 E | JF318097 |
| Niez7  Poland | *Hyla arborea* | - | Poland, Niezgoda | 51.5153 N  17.0508 | JF318098 |
| Niez8  Poland | *Hyla arborea* | - | Poland, Niezgoda | 51.5153 N  17.0508 E | JF318099 |
| Niez9_  Poland | *Hyla arborea* | - | Poland, Niezgoda | 51.5153 N  17.0508 E | JF318100 |
| NME1228_0  Germany | *Hyla arborea* | NME A 1228a/04 | Germany, Thüringen, TÜP Egstedt, Erfurt | 50.9235 N  11.0688 E | FJ226869 |
| NME674_00  Croatia | *Hyla arborea* | NME A 0674/00 | Croatia, Cres island, Osor, pond near transformation plant | 44.690 N  14.390 E | FJ226870 |
| NME675_00  Croatia | *Hyla arborea* | NME A 0675/00 | Croatia, Insel Cres, Lubenice, pond in the NE | 44.880 N  14.330 E | FJ226922 |
| NME676_00  Croatia | *Hyla arborea* | NME A 0676a/00 | Croatia, Cres island, Hrasta, Salix row near the village | 44.810 N  14.410 E | FJ226871 |
| NME902_01  Greece | *Hyla arborea* | NME A 0902a/01 | Greece, Peloponnes, E bank of Stymphalian lake, Kiónia | 37.850 N  22.450 E | FJ226783 |
| NME902_01  Greece | *Hyla arborea* | NME A 0902b/02 | Greece, Peloponnes, E bank of Stymphalian lake, Kiónia | 37.850 N  22.450 E | FJ226784 |
| RoFo1  Romania | *Hyla arborea* | - | Romania, Foieni | 47.4252 N  22.1956 E | JF318101 |
| RoFo10  Romania | *Hyla arborea* | - | Romania, Foieni | 47.4252 N  22.1956 E | JF318102 |
| RoFo16  Romania | *Hyla arborea* | - | Romania, Foieni | 47.4252 N  22.1956 | JF318103 |
| RoFo5  Romania | *Hyla arborea* | - | Romania, Foieni | 47.4252 N  22.1956 E | JF318104 |
| RoRe1  Romania | *Hyla arborea* | - | Romania, Resighea | 47.3679 N  22.1616 E | JF318105 |
| RoRe10  Romania | *Hyla arborea* | - | Romania, Resighea | 47.3679 N  22.1616 E | JF318106 |
| RoRe13  Romania | *Hyla arborea* | - | Romania, Resighea | 47.3679 N  22.1616 E | JF318107 |
| RoRe5  Romania | *Hyla arborea* | - | Romania, Resighea | 47.3679 N  22.1616 E | JF318108 |
| Szcz_1  Poland | *Hyla arborea* | - | Poland, Szczodre | 51.2044 N  17.2056 E | JF318109 |
| Szcz_2  Poland | *Hyla arborea* | - | Poland, Szczodre | 51.2044 N  17.2056 E | JF318110 |
| Szcz_3  Poland | *Hyla arborea* | - | Poland, Szczodre | 51.2044 N  17.2056 E | JF318111 |
| Szcz_4  Poland | *Hyla arborea* | - | Poland, Szczodre | 51.2044 N  17.2056 E | JF318112 |
| Szcz_5  Poland | *Hyla arborea* | - | Poland, Szczodre | 51.2044 N  17.2056 E | JF318113 |
| Szcz_6  Poland | *Hyla arborea* | - | Poland, Szczodre | 51.2044 N  17.2056 E | JF318114 |
| Szcz_7  Poland | *Hyla arborea* | - | Poland, Szczodre | 51.2044 N  17.2056 E | JF318115 |
| Szcz_8  Poland | *Hyla arborea* | - | Poland, Szczodre | 51.2044 N  17.2056 | JF318116 |
| Szcz_9  Poland | *Hyla arborea* | - | Poland, Szczodre | 51.2044 N  17.2056 | JF318117 |
| Z187  Belgium | *Hyla arborea* | - | Belgium, Zenhoven, Wijvenheide | 50.9871 N  5.3672 | JF318118 |
| Z194  Belgium | *Hyla arborea* | - | Belgium, Zenhoven, Wijvenheide | 50.9871 N  5.3672 E | JF318119 |
| Z203  Belgium | *Hyla arborea* | - | Belgium, Zenhoven, Wijvenheide | 50.9871 N  5.3672 E | JF318120 |
| Z211  Belgium | *Hyla arborea* | - | Belgium, Zenhoven, Wijvenheide | 50.9871 N  5.3672 E | JF318121 |
| IG71  Switzerland | *Hyla intermedia* | - | Switzerland, Grangette | 46.39 N  6.9 E | FJ226857 |
| IPA12M  Switzerland | *Hyla intermedia* | - | Switzerland, Piazzogna | 46.1362 N  8.8206 E | JF318122 |
| IPA13M  Switzerland | *Hyla intermedia* | - | Switzerland, Piazzogna | 46.1362 N  8.8206 E | JF318123 |
| IPA2F  Switzerland | *Hyla intermedia* | - | Switzerland, Piazzogna | 46.1362 N  8.8206 E | JF318124 |
| IPA6F  Switzerland | *Hyla intermedia* | - | Switzerland, Piazzogna | 46.1362 N  8.8206 E | JF318125 |
| NME916_01  Italy | *Hyla intermedia* | NME A 0916a/01 | Italy, Calabria, Paola, Villaggio Bahja | 39.350 N  16.033 E | FJ226877 |
| Sicily81  Italy | *Hyla intermedia* | - | Italy, Sicily, coastal Pond E of Capo d'Orlando | 38.1176 N  14.6988 E | FJ226881 |
| Sicily101  Italy | *Hyla intermedia* | - | Italy, Sicily, near Mazarra di Sicilia | 380960 N  15.1384 E | FJ226878 |
| Sicily102  Italy | *Hyla intermedia* | - | Italy, Sicily, Tindary Peninsula, coastal pond | 38.1396 N  15.0526 E | FJ226879 |
| Sicily103  Italy | *Hyla intermedia* | - | Italy, Sicily, San Leonardo River | 37.3342 N  15.0804 E | FJ226880 |
| Ti1  Switzerland | *Hyla intermedia* | - | Switzerland, Tessin | 46.1361 N  8.8204 E | FJ226853 |
| Ti2  Switzerland | *Hyla intermedia* | - | Switzerland, Tessin | 46.1361 N  8.8204 E | FJ226852 |
| HmNew1  France | *Hyla meridionalis* | - | France, Camargue, Tour du Valat | 43.52 N  4.7 E | FJ226924 |
| HPA248  Portugal | *Hyla molleri* | - | Portugal (248), north from Marvao, Beira village | 39.4510 N  7.3597 W | FJ226917 |
| M_33  Spain | *Hyla molleri* | - | Spain, Valdemanco | 40.8531 N  3.6451 W | JF318126 |
| M_34  Spain | *Hyla molleri* | - | Spain, Valdemanco | 40.8531 N  3.6451 W | JF318127 |
| M_M  Spain | *Hyla molleri* | - | Spain, south of Salamanca | 40.9616 N  5.6676 W | FJ226918 |
| M01  Spain | *Hyla molleri* | - | Spain, Toledo province, near Almorox | 40.31684.3804 W | JF318128 |
| M02  Spain | *Hyla molleri* | - | Spain, Avila province, Camino Real de El Tiemblo | 40.41134.4683 N | JF318129 |
| M03  Spain | *Hyla molleri* | - | Spain, Avila province, Camino Real de El Tiemblo | 40.4113 N  4.4683 W | JF318130 |
| M04  Spain | *Hyla molleri* | - | Spain, Avila province, Camino Real de El Tiemblo | 40.4113 N  4.4683 W | JF318131 |
| M05  Spain | *Hyla molleri* | - | Spain, Avila province, Camino Real de El Tiemblo | 40.4113 N  4.4683 W | JF318132 |
| M06  Spain | *Hyla molleri* | - | Spain, Avila Province, El Hoyo de Pinares | 40.4860 N  4.3897 W | JF318133 |
| MNCN  ADN3448  Spain | *Hyla molleri* | MNCN  3448 | Spain, Madrid Charcas de Miraflores, Parque Natural de Peñalara, Rascafría | 40.8476 N  3.9486 W | JF318134 |
| MNCN  ADN11068  Spain | *Hyla molleri* | MNCN11068 | Spain, La Coruña, Embalse de Cecebre Cambre | 43.2745 N  8.2821 W | JF318135 |
| MNCN  ADN11078  Spain | *Hyla molleri* | MNCN  11078 | Spain, Burgos, Laguna de Pilavieja, merindad del río Ubierna. Cernégula | 42.6388 N  3.6239 W | JF318136 |
| MNCN  ADN11086  Spain | *Hyla molleri* | MNCN  11086 | Spain, Avila, Carretera de Candeleda a Navalcán, 2 km from Candeleda | 40.1262 N  5.2206 W | JF318137 |
| WP200A  Spain | *Hyla molleri* | WP200A | Spain, Guadalajara, La Mierla | 40.9233 N  3.2609 W | JF318138 |
| WP205A  Spain | *Hyla molleri* | WP205A | Spain, Madrid, Guadalix de la Sierra | 40.7555 N  3.6761 W | JF318139 |
| WP207A  Spain | *Hyla molleri* | WP207A | Spain, Madrid, El Berrueco | 40.9319 N  3.5692 W | JF318140 |
| WP208A  Spain | *Hyla molleri* | WP208A | Spain, Madrid, Colmenar Viejo | 40.6871 N  3.8299 W | JF318141 |
| WP211A  Spain | *Hyla molleri* | WP211A | Spain, Madrid, Cerceda | 40.7187 N  3.9562 W | JF318142 |
| WP212A  Spain | *Hyla molleri* | WP212A | Spain, Madrid, Puerto de Canencia | 40.8712 N  3.7565 W | JF318143 |
