# Supplementary material for: Ever-Young Sex Chromosomes in European Tree Frogs
Source: PLoS Biol. 2011 May 17;9(5):e1001062. doi: 10.1371/journal.pbio.1001062 (PMC3100596; doi:10.1371/journal.pbio.1001062)
Supplement: Text S1 — Sex differences in allelic frequencies. (DOC) [file pbio.1001062.s007.doc]

## Text S1

**Sex-specific differences in allelic frequencies.**

In *H. arborea*, several loci from the linkage group under focus show sex-diagnostic alleles. In the *Lavigny* population (13 mating pairs sampled), allele *236* at locus *Ha* 5-22 occurs only in males, with one copy each (Table S3a). From combinatorial statistics, the probability that this occurs by chance, assuming autosomal localization, is obtained as the ratio of $\frac{13!}{13!0!}2^{13}$ (number of combinations of 13 copies of allele *236* among 13 males, one copy each) over $\frac{52!}{13!39!}$ (number of combinations of these 13 copies among 52 copies of genes), which amounts to p = 1.29 10^-8^.

In *H. molleri*, at locus *Ha* 5-22, four out of the five males from the *Valdemanco* population harbor one and only one copy of allele *239* (not found in any of the five females). The probability that this happens by chance (assuming autosomal localization) is obtained as the ratio of $\frac{5!}{4!1!}2^{4}$ (number of combinations of four copies of allele *239* among five males, maximum one copy each) over $\frac{20!}{4!16!}$ (number of combinations of these four copies among 20 copies of genes), which amounts to *p* = 0.0165.

In *H. intermedia*, allele *221* at locus *Ha* 5-22 was significantly more frequent in males than in females (Table S3b; *q* = 0.73 vs 0.42; χ^2^ = 9.58; p<0.001). The same was true for allele *250* at locus *Ha* H-108 (*q* = 0.77 vs 0.46; χ^2^ = 9.89; p<0.001). Sibship analyzes actually show this allele *250* to be fixed on the Y, and to segregate at frequency *q* = 0.49 on the X. Accordingly, all of the seven adults (out of 48) with no copy of allele *250* were females. Under the assumption of autosomal localization, the probability that such a sex-bias arises by chance is calculated as the ratio of $\frac{24!}{7!17!}$ (number of combinations of seven individuals among 24 females) over $\frac{48!}{7!41!}$ (number of combinations of seven individuals among 48 adults), which amounts to p< 0.005.

In this same *H. intermedia* population, at locus *Ha* D-110 a null allele was found at frequency 0.44 in males and 0.88 in females (Table S3b; χ^2^ = 20.36, p<0.0001). All of the 19 individuals homozygous for this null allele were females. The probability that such as sex bias arises by chance, calculated as the ratio of $\frac{24!}{19!5!}$ over $\frac{48!}{19!29!}$, amounts to *p* = 3.67 10^-9^. The obvious parsimonious alternative is that the null allele segregates only on the X, as otherwise supported by sibship analyzes.
