# Supplementary material for: Ever-Young Sex Chromosomes in European Tree Frogs
Source: PLoS Biol. 2011 May 17;9(5):e1001062. doi: 10.1371/journal.pbio.1001062 (PMC3100596; doi:10.1371/journal.pbio.1001062)
Supplement: Text S2 — Non-recombining Y haplotypes in males. (DOC) [file pbio.1001062.s008.doc]

## Text S2

**Non-recombining Y haplotypes in males**

In the *Lavigny* populations of *H. arborea*, sibship analyzes allowed identifying four non-recombining haplotypes, segregating in the 13 males with frequencies 0.54, 0.23, 0.15, and 0.08 respectively (Table S3a). These haplotypes have fixed at all loci the same allele (not found in females), except for *Ha* 1-60 (where four alleles segregate, none of them found in females) and *Ha* H-107 (allele *267* also found in females), and thus appear recently diverged from a common Y ancestor, but more anciently from the X.

In the *Piazzogna* population of *H. intermedia*, combining sibship analyzes with sex-specific allelic frequencies allowed identifying five haplotypes (over the six loci genotyped) segregating among the 24 males sampled from this population, with frequencies 0.46. 0.21, 0.17, 0.12 and 0.04 respectively (Table S3b). All males with allele *Ha* A-103*/223* also had allele *Ha* D-110*/485*. Reciprocally, the eight males with alleles *Ha* D-110*/519* or *528* also had allele *Ha* A-103/*227* (otherwise found on the *X* copy of one additional male). The probability that such association is found by chance, assuming these markers to be on an autosome (and therefore to recombine in females), is calculated as the ratio of $\frac{9!}{8!1!}$ (number of combinations of eight males with allele *519* or *528* among the nine males with allele *227*) over $\frac{24!}{16!8!}$ (total number of combination of these eight individuals among 24 males), i.e. *p* = 1.22 10^-5^.

In *H. molleri*, among the 13 males genotyped from the *Cantera* population, we identified a minimum of two markedly different haplotypes (7 and 6 individuals each) with different alleles fixed at loci *Ha* 5-22, *Ha* M3, *Ha* 1-60 and *Ha* A-103 (Table S3c). The seven individuals assigned to the same haplotype display one copy (and only one) of alleles *Ha* M3*/170* and *Ha* A-103*/235* (otherwise not found in any other individual). The probability that this occurs by chance, assuming autosomal localization, is obtained as the ratio of $\frac{13!}{6!7!}2^{14}$ (number of combinations for which the alleles *170* and *235* are found only in males, same seven individuals, one copy each) divided by (total number of combinations assigning the seven copies of *Ha* M3*/170* and the seven copies of *Ha* A-103*/235* among the 15 diploid individuals, independent of sex) which amounts to *p* = 6.78 10^-6^.
